# Modification of Monolignol Biosynthetic Pathway in Jute: Different Gene, Different Consequence

**DOI:** 10.1038/srep39984

**Published:** 2017-01-04

**Authors:** Farhana Shafrin, Ahlan Sabah Ferdous, Suprovath Kumar Sarkar, Rajib Ahmed, Al- Amin, Kawsar Hossain, Mrinmoy Sarker, Jorge Rencoret, Ana Gutiérrez, Jose C. del Rio, Neeti Sanan-Mishra, Haseena Khan

**Affiliations:** 1 Molecular Biology Laboratory, Department of Biochemistry and Molecular Biology, University of Dhaka, Dhaka-1000, Bangladesh; 2 Dept. Plant Biotechnology IRNAS-CSIC P.O. Box 1052, 41080-Seville, Spain; 3 Plant RNAi Biology Group, International Center for Genetic Engineering and Biotechnology (ICGEB), New Delhi-11006, India

## Abstract

Lignin, a cross-linked macromolecule of hydrophobic aromatic structure, provides additional rigidity to a plant cell wall. Although it is an integral part of the plant cell, presence of lignin considerably reduces the quality of the fiber of fiber-yielding plants. Decreasing lignin in such plants holds significant commercial and environmental potential. This study aimed at reducing the lignin content in jute-a fiber crop, by introducing hpRNA-based vectors for downregulation of two monolignoid biosynthetic genes- cinnamate 4-hydroxylase (*C4H*) and caffeic acid *O*-methyltransferase (*COMT*). Transgenic generations, analyzed through Southern, RT-PCR and northern assays showed downregulation of the selected genes. Transgenic lines exhibited reduced level of gene expression with ~ 16–25% reduction in acid insoluble lignin for the whole stem and ~13–14% reduction in fiber lignin content compared to the control lines. Among the two transgenic plant types one exhibited an increase in cellulose content and concomitant improvement of glucose release. Composition of the lignin building blocks was found to alter and this alteration resulted in a pattern, different from other plants where the same genes were manipulated. It is expected that successful *COMT*-hpRNA and *C4H*-hpRNA transgenesis in jute will have far-reaching commercial implications leading to product diversification and value addition.

Growing environmental awareness together with a gradual curb of fossil fuels has led to a paradigm shift in the search for new fuel reserve and has recognized the potential of living materials as a source of renewable energy. Bioethanol, produced from biomass, is considered a promising transportation fuel, for which the major plant types used are sugarcane and corn^1^. However, bioethanol production from these crops has its limitations since they provide little or no net life cycle benefit in terms of carbon dioxide and other greenhouse gas (GHG) emission reductions^2^ and also because of their recognizable incorporation in our food supply chain^3^. On the other hand, lignocellulose biomass obtained from agricultural remains, forest residues (hardwood and softwood), herbaceous and woody energy crops- is renewable, cheap, adaptable to less cultivable land and readily obtainable with 10–50 billion tons produced per year at the global level^4^. It has therefore been considered an attractive replacement of food-crop based source of biofuels^2^. Lignocellulosic biomass is also a potential source of raw materials for the paper and pulp industry^5^.

Nevertheless, the popularity of lignocellulose as an alternative source of biofuel, paper and pulping industry is severely hindered by its structural features. Crystalline cellulose embedded in a hemicellulose and lignin matrix, renders enzymatic, microbial or chemical conversion of cellulose to glucose difficult^6^. Existing biomass fermentation procedures for fuels and chemicals are generally expensive principally because of this recalcitrance, which in turn lead to limited commercialization of bioethanol^7^. Recent plant genetic engineering studies have aimed to decrease the cost by lowering physical hindrance of plant cell wall and to increase the cellulose and/or overall crop biomass yield^8^. Accessibility of plant cell wall polysaccharides to digestion is limited by many factors, including the presence of lignin, a phenylpropanoid polymer in vascular tissues and fibers^9^. Attempts have been made to lower the lignin content and/or change the composition by reducing the need for expensive and harsh pretreatments required to disintegrate the same and to allow access to cellulose^10^. There has also been an intense focus of research to alter lignocellulosic materials currently used in paper as well as pulp industries in order to improve pulp production, reduce the amount of energy required and the environmental pollution associated with biomass processing^11^.

Jute (*Corchorus sp*.), a lignocellulosic annual plant grown abundantly in Bangladesh, India, China and Thailand, is known for its fiber quality and is widely used for the manufacture of flexible packing fabrics, carpet backings, decorative fabrics, automotive headlining and other applications^12^. Agro-based nature, annually renewable, biodegradable and obtainable at a low cost put jute at an advantage^13^. Biodegradable nature of jute fiber and the potential high yield of cellulose biomass per acre have increased the global interest in jute. Textile and paper industries are also interested in its potential as an important ingredient for producing paper and fine textiles^14^. As with other lignocellulosic plants the drawback of jute when used as a source for such purposes is the abundance of lignin polymer that renders the plant material almost inaccessible for downstream processes. The high amount of lignin in jute makes it coarse and impedes use of the same as a textile fiber.

In this context, the current study aimed at modifying the lignin biosynthetic pathway of jute (*Corchorus olitorius* var. O-9897) for induced altered lignin composition, increase in cellulose content and enzymatic digestibility. This was attempted by downregulating two major genes, cinnamate 4-hydroxylase (*C4H*) and caffeic acid 3-*O* methyltransferase (*COMT*) of the lignin biosynthetic pathway by using the technique of RNAi^15^. Ten different enzymes catalyze a series of hydroxylation, methylation and side chain reduction reactions of monolignol precursors^16^. C4H is involved near the beginning of the lignin pathway which hydroxylates cinnamic acid to form *p*-coumaric acid^17^. COMT functions late in the monolignol biosynthetic pathway and, despite its name, methylates 5-hydroxyconiferyl aldehyde and 5-hydroxyconiferyl alcohol to form S unit precursors, sinapyl aldehyde and sinapyl alcohol, respectively^18^ These two genes, *COMT* and *C4H* do not appear to overlap with the plant defense machinery^19^. Transgenic jute lines with reduced expression of *COMT* and *C4H* genes were found to have lowered lignin content, altered lignin composition, increase in total cellulose content and improvement, albeit small in enzymatic saccharification. Moreover, none of these alterations led to any growth compensation. The results bolster the potential of lignin modification as a tool for improving lignocellulosic crops like jute so that it may be used more efficiently as a source of biofuel, paper pulp and textile fiber.

## Results

### Gene identification, construction of RNAi vector and validation

Full length sequences of each selected gene namely, caffeic acid *O*-methyltransferase (*COMT*) and cinnamate 4-hydroxylase (*C4H*) in jute were retrieved by using traditional gene walking method with degenerate primers designed from plants with sequence similarity to jute like *Populus, Vitis, Gossypium* etc^20^. This was followed by cloning and sequencing of the same.

Successful *COMT*-hpRNA and *C4H*-hpRNA constructs were designed from the conserved region of the respective genes (details given in methods). A set of construct specific PCRs revealed an inverted orientation of the two copies of *COMT*-hpRNA and *C4H*-hpRNA sequences. Determination of full-length sequence of the recombinant destination vectors (see methods) further validated the same (Supplementary Fig. 1.1, 1.2).

### Transformation of jute plants

**Figure 1:**
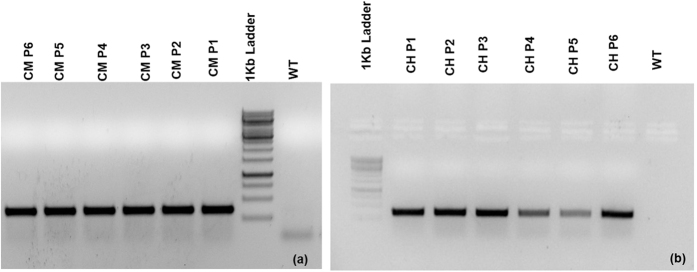
Confirmation of initial transgenesis. Genomic DNA isolated from randomly selected transformed plants was PCR amplified with *NPT(II*) primer for *COMT*-hpRNAi (**a**) and *C4H*-hpRNAi (**b**) lines to confirm the initial transgenesis.

An *in planta* protocol for jute transformation developed by Sajib *et al*.^21^ was used to introduce the *COMT*-hpRNA and *C4H*-hpRNA based constructs into *C. olitorius* var 0–9897 independently to give rise to two different groups of transgenic lines (*COMT*-hpRNA and *C4H*-hpRNA lines). Initial confirmation of transgenesis was done by amplification of a region of *NPT(II*) (neomycin phosphotransferase II, determinant of kanamycin resistance) reporter gene using genomic DNA isolated from the leaves of randomly selected transgenic plants from each group and wild type control. PCR results showed prominent presence of the reporter gene in transgenic plants. No signal was obtained for the wild type control. Among the positive transgenic plants, six chosen arbitrarily for further analysis were designated CM P1 -P 6 for *COMT*-hpRNA and CH P1 - P6 for *C4H*-hpRNA plant lines (Fig. 1a,b).

**Figure 2:**
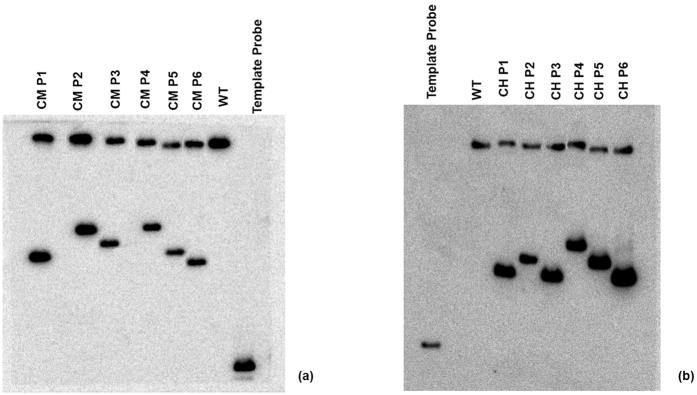
Southern analysis of primary hpRNAi transformants of jute. Blots were probed with [α^32P^] labeled hpRNAi construct specific region of different (**a**) *COMT*-hpRNA and (**b**) *C4H*-hpRNA transgenic plants.

Successive T1 generations of each line mentioned above were screened by their growth on media containing 400 mM kanamycin, the resistance gene for which, is present in the RNAi vector. For both the genes ~75% of the assumed transformants were able to grow in the antibiotic media, indicating efficient transformation. Seedlings tested positive were used for subsequent molecular studies. Southern blot analysis was performed for T1 plants of the six lines of each transgenic type to further confirm the incorporation of hpRNA for both genes in the jute genome. Single conspicuous bands were detected in the blotting experiments when hpRNA precursor specific probes were used (Fig. 2a,b).

### Downregulation of *COMT* and *C4H* genes in jute

**Figure 3:**
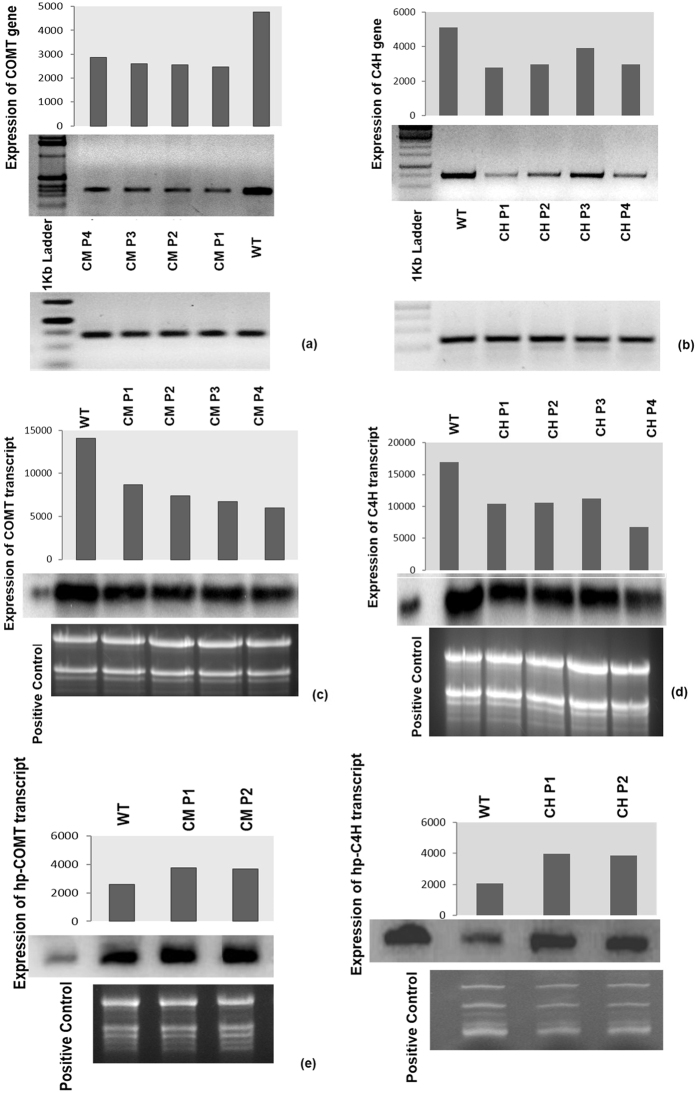
Expression analysis of downregulated genes as a proof of functionality of the RNAi construct. RT-PCR of *COMT* (**a**) and *C4H* (**b**) gene along with actin gene as loading control. Northern blot with [α^32P^] labeled cDNA probe of *COMT* (**c**) and *C4H* gene (**d**). For hp-northerns, the probes were hpRNAi amplicon specific sequences of *COMT* (**e**) and *C4H* (**f**) genes respectively. The figures show intensities of bands normalized with respect to 28S rRNA.

Among these six lines of each transgenic type found positive by Southern analysis, four (CM P1-P4 and CH P1-P4) from each group were then considered for assessing the functionality of the hpRNA constructs. The hpRNA for *COMT* and *C4H* appeared to be effective in activating the RNAi pathway leading to depleted gene expression. Expression was found to drop in comparison to the wildtype for both genes when monitored by semi-quantitative RT-PCR (Fig. 3a,b). Also, in the northern blots, intensities of the bands for expressed genes were found to be lower in comparison to non-transgenic plants for both lines, representing suppression of the respective genes and hence validating the functionality of the hpRNA constructs (Fig. 3c,d). Further experiments were conducted for T1, T2 and T3 generations of CM P1-P2 as well as CH P1-P2 plants. Each experiment (measurement of lignin and cellulose content and the amount of glucose released) was conducted taking into account biological duplicates with three technical replicates. Representative lines were also subjected to hpRNA northern blot analysis (Fig. 3e,f) to check their expression levels in transgenic plants. The blot showed an opposite trend of expression confirming successful and functional hpRNA transgenesis for both the genes. Signals were also observed in wild type samples because the hpRNAs were designed from endogenous *COMT* and *C4H* genes of *C. olitorius*. However, the intensities were much higher in transgenic lines in comparison to the wild type.

### Phenotypical and histochemical analysis

Morphological assessments in terms of plant height, width, pod number and average pod length of all plants grown under field conditions were compared (Table 1) in order to determine if there were any differences in the vegetative and reproductive stages of the transformed jute generations. Plants were randomly chosen from the selected lines of each group. No distinct morphological variations with respect to wild type plants were observed. Plant height, width and pod length were considerably same in control and each type of transgenic plant generations (p = 0.3, 0.037 and 0.058 respectively). Random variations in the number of pods (ranging from 58–63) were seen in both control and different transgenic plant types. This therefore cannot be attributed to any effect of gene downregulation.

**Figure 4:**
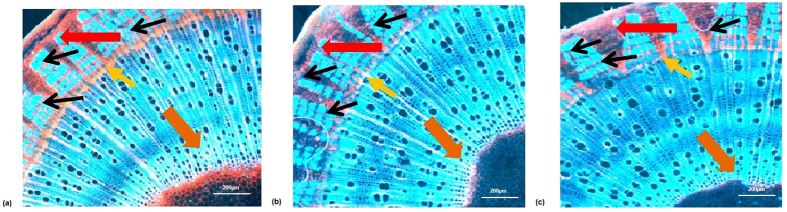
Histochemical assay of lignin. Lignin deposition observed in wildtype plants (**a**) and transgenic lines *COMT*-hpRNA (**b**) and *C4H*-hpRNA (**c)**. The arrows indicate difference in lignin deposition in transgenic and wild type jute plants (red: epidermis; orange: pith; yellow: vascular cambium). The area between the epidermis and vascular cambium (indicated by black arrows) is the bast region from where the jute fiber is obtained. This area as observed is significantly less lignified in transgenic plants than the wild type.

Histochemical analysis of stem cross sections of wild type and T3 generations of the selected plant lines was performed using phloroglucinol (a dye which specifically stains native lignin due to the formation of a purple to reddish chromophore between lignin hydroxycinnamaldehyde end-groups and phloroglucinol), showed significantly lower deposition of lignin in both transgenic type. This is indicated by a reduction in the staining intensity when compared to control plants (Fig. 4). Variation in epidermal lignin content was not much for the different plant types but decreased lignin accumulation was observed in the phloem fiber (the region from where jute fiber is obtained) and the cortex of the transgenic lines compared to the wild type. The vascular cambium ring of each transgenic type was almost devoid of lignin. Within these plants the parenchymal tissue of the pith region, showed significant reduction of lignin deposition in comparison to the wild type. The overall lignin content appeared to be lower for *C4H*-hpRNA than that of *COMT*-hpRNA lines which supported the value obtained for Klason lignin (Table 2).

### Lignin content and composition

**Figure 5:**
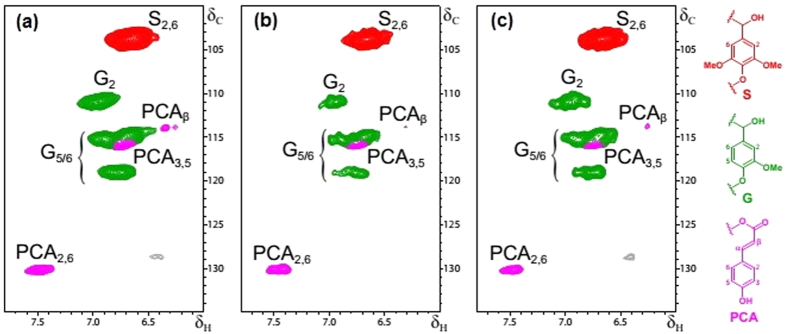
2D-HSQC-NMR spectra of the aromatic/unsaturated regions from whole jute stems representative of (**a**) wild type plants, (**b**) *C4H*-hpRNA lines, and (**c**) *COMT*-hpRNA lines. Main cross-signals in the aromatic/unsaturated region (δ_C_/δ_H_ 100–135/6.0–7.8) of the HSQC spectra corresponded to the aromatic rings and unsaturated side-chains of the different guaiacyl (G) and syringyl (S) lignin units, and to *p*-coumarates (PCA) that are associated to the lignin. Main lignin units detected in the aromatic region of the 2D HSQC NMR spectra of jute plants are displayed in the right side: G, guaiacyl units; S, syringyl units; PCA, *p*-coumarates.

To estimate if the lignin content was reduced as an effect of downregulation of genes under consideration after incorporation of hpRNA, amount of lignin was estimated by the Klason lignin method. For measuring lignin reduction, three generations of the two lines (CM P1-P2 and CH P1-P2) of each transgenic plant type were taken into consideration and compared to the control. Samples used were randomly selected ensuring two biological replicates with technical triplicates of T1 - T3 generations. Different constituents of lignin were determined by 2D NMR spectroscopy (Fig. 5). Substantial decrease in the lignin content was found for both transgenic type in comparison to the control. On an average, 16% reduction in the lignin content was found for the whole stem of the *COMT* -hpRNA- line and about 13.5% for the fiber alone when compared with wild type data. These *COMT*-hpRNA lines showed almost similar S/G ratio to that of the wild type. As for *C4H* -hpRNA- line, lignin reduction was calculated to be about 22% for the whole stem and an average of 14% for the fiber. The S/G ratio was found to increase in these lines in comparison to the wild type. Over all, *C4H*-hpRNA lines showed more decrease in lignin content than the *COMT*-hpRNA lines. S/G ratios were considerably different, up to the T2 generations for both transgenic plant types. However, T3 generations of these plants had an S/G ratio similar to that of the wild type.

### Effect on cellulose content

In our study, both types of transgenic lines showed a considerable increase in the cellulose content in all three generations. This increase was up to 4% for *COMT-* hpRNA lines compared to the wild type with a concomitant increase in cellulose-to-lignin ratio (Table 2). In *C4H-* hpRNA lines, cellulose content on an average increased 3.5%, together with an increase in cellulose/lignin ratio. Modification induced by reduced expression of *COMT*- led to substantially more accumulation of cellulose than lines with reduced *C4H* expression. However, cellulose-to-lignin ratio was better in *C4H-* hpRNA, because of the lower lignin content than that of *COMT-* hpRNA lines.

### Reduced recalcitrance in transgenic plants

Efficiency of glucose release (as a parameter of reduced recalcitrance) was measured for the transgenic lines. With reduced *C4H* transcript level, considerably more glucose was released (an increase of about 2%) from these transgenic lines than the wild type (Table 2). However, for *COMT*-hpRNA lines the amount of glucose release was almost the same as the control.

## Discussion

The current trend of altering lignin biosynthetic pathway with a view to reducing the amount of lignin is largely effective in enhancing the profitable usability of lignified plant-based resources. Jute, a ligno-cellulose-rich fiber crop, delineates a new sphere with respect to lignin manipulation. In the light of several literature reports^5^,^22^,^23^, it can be assumed that lignin engineering can reinforce the use of jute as a sustainable source of bio-based materials for commercial purposes (viz. textile, paper and pulping, biofuel). This study emphasized on reducing the lignin content in jute, using the RNAi technique (siRNA) for fine-tuned alteration of the lignin biosynthetic pathway.

RNA interference (RNAi) is based on sequence-specific RNA degradation that follows formation of double-stranded RNA (dsRNA) homologous in sequence to the target gene^24^. RNAi vector mediated gene silencing has increased the efficiency of genetic manipulation in plants because of its ease of application and possibilities for genome-wide reverse genetics^25^. With the advent of gene silencing techniques, many studies have shown that efficient repression of target genes can be induced by expressing self-complimentary hpRNA constructs, known as hpRNAi^26^. Gene constructs encoding intron spliced RNA with a self-complementary hairpin (hp) structure are known to induce post-transcriptional gene silencing with almost 100% efficiency when directed against viruses or endogenous genes and transgenes^27^. Because the method of RNAi is particularly sequence specific, elucidation of the very sequence of jute genes was imperative. Due to unavailability of jute genome sequence in public database, first challenge of this study was to retrieve the full length sequence of the selected genes. Traditional method of gene walking was found to be effective because of sequence similarity of these genes among different species.

Lignin content and composition are two aspects that contribute to recalcitrance of a cell wall^28^. Among plants the lignin content can vary from 15% to 40%^29^. Deposition of lignin in cell walls is a crucial step in the adaptation of plants to a land habitat, as such, plants tolerate up to 40% reduction in lignin without major adverse effects on normal plant growth and development in greenhouse conditions^30^. Lignin is measured by the Klason method which determines the total residue remaining after removal of cell wall polysaccharides by sulfuric acid^31^. We observed significant reduction in lignin content in both types of transgenic plants (*C4H*-hpRNA and *COMT*-hpRNA lines) (Table 2). Effect of down regulating *COMT* expression has previously been reported for transgenic lines of tobacco^23^, maize^32^, alfalfa^33^, switchgrass^34^ etc. in which down regulation of *COMT* expression was found to accompany a reduction in the lignin amount, consistent with our findings. Decreasing *C4H* gene expression in tobacco^35^, alfalfa^6^ and eucalyptus^36^ showed a similar reduction in the lignin content.

Lignin is composed of a combination of sinapyl (S), coniferyl (G), and 4-coumaryl (H) alcohol subunits which polymerize with each other^10^. Composition of lignin varies widely based on plant species and subunit availability. Relative levels of S, G, and H lignin subunits are expressed as the S/G/H ratio. Since, H subunits are in insignificant amount in woody species^37^ S/G ratio is therefore the general expression of lignin subunits. In addition to the three main monolignols, lignin contains small amount of units from incomplete monolignol biosynthesis and integrates various other phenylpropanoid units, such as hydroxycinnamyl aldehydes, acetates, *p*-coumarates, *p*-hydroxybenzoates, and tyramine ferulates^38^. Since the polymerization of monolignols is random, lignin composition is highly plastic and amenable to manipulation^39^. Lignins rich in G units has relatively more carbon-carbon bonds than lignin rich in S lignin, as a consequence, lignins essentially made of G units are less susceptible to delignification than lignins made of S units^40^. Changes in lignin monomer composition are most readily visualized from the aromatic regions of two-dimensional NMR (HSQC) ^1^H–^13^C correlation signals^41^. Increasing the S-to-G ratio by altering lignin biosynthesis related gene expression can make a cell wall somewhat easier to degrade^42^.

The S/G ratio in this study was found to increase in *C4H*-hpRNA lines as opposed to other experiments of downregulation of *C4H* gene in tobacco^35^, alfalfa^6^ and eucalyptus^36^, in which *C4H* downregulation showed a drop in S/G ratio. The discrepancy between *C4H* downregulation and corresponding change in S/G ratio has been enigmatic to researchers. Because of its relative position in the pathway, the S/G ratio is expected to rise when *C4H* expression is lowered. In contrast to other reports artificial downregulation of *C4H* in jute supported the expected rise in the S/G ratio. The increase in S/G ratio in the transgenic lines of *C4H*-downregulated plants is due to an increase in the S lignin level (Table 2). Differences in lignin composition in *C4H*-hpRNA transgenic lines in jute and other plants cannot be linearly described because of the metabolic complexity of lignin biosynthetic pathway. Although this pathway has been studied for more than a century now, there are still labyrinths that are not fully understood^9^. Variation in S/G lignin ratio as an effect of downregulation of *C4H* in jute and other plants may not be purely the result of a change in the overall fluctuation of the pathway determined by the extent of downregulation of the individual enzymes. Metabolic channeling may function at different stages of the phenylpropanoid biosynthetic pathway^43^, although there is no direct confirmation yet of the presence of separate metabolic channels leading to G and S lignin^44^. It is also possible that there are isoforms of *C4H* present in jute as reported for oilseed rape^45^, orange^46^, poplar^47^ and white popinac^48^ which influence the metabolic steps of the pathway. Recently a predictive kinetic metabolic-flux (PKMF) model has been proposed and validated in poplar which shows that two paralogs of *C4H* are kinetically distinct with different Km and Kcat values and contribute differentially to the metabolic-flux converting cinnamic acid to 4-coumaric acid; this model with experimental validations showed that a reduction in *C4H* expression, resulted in low lignin and a high S/G ratio^49^. An increase in *p*-coumarate in transgenic generations of *C4H*-hpRNA line (Table 2) suggests that although *C4H*, responsible for biosynthesis of *p*-coumarate is down-regulated, a second pathway responsible for altering lignin composition could possibly be active.

An increase in the amount of cellulose and a doubled cellulose to lignin ratio have been reported for most severely lignin-reduced transgenic trees of aspen^50^ and poplar^51^. In our study, the transgenic lines showed an increased level of cellulose content (Table 2). This might be due to relative locations of the enzymes in the lignin biosynthetic pathway which influence the associated surge in cellulose content because it is assumed that, cellulose synthesis is normally substrate limited and reducing the flow at different stages of the lignin pathway increases the availability of carbon for cellulose deposition^50^.

Reduced recalcitrance may be referred to as an escalation in sugar release after enzymatic saccharification relative to the control samples under a defined pretreatment condition. In other words, it is an increase in the digestibility of polysaccharide components to liberate sugar^22^. There are many reports indicating that biomass digestibility is negatively correlated with lignin concentration. Genetic and transgenic approaches to manipulate these parameters have resulted in varying degrees of success with regard to improving cell wall digestibility^6^,^52^. Caffeic acid 3-*O*-methyltransferase (*COMT*) downregulation has led to increased saccharification in tobacco^53^, sorghum^54^, alfalfa^33^ and switchgrass^34^. Positive effect of *C4H* downregulation in saccharification and glucose release has also been reported in eucalyptus. For jute in our case, the amount of glucose released from the different transgenic plants (*C4H*-hpRNA and *COMT*-hpRNA lines) may be attributed to S/G ratios of the same. S/G ratio influences biomass digestibility with S lignin being favorable for hydrolysis because of less crosslinking compared to G lignin^53^. Between the two, *C4H*-hpRNA lines showed a decrease in lignin content, increase in S/G ratio and consequently better sugar release compared to *COMT*-hpRNA lines which showed a drop in the S lignin content. However, improvement in digestibility was not as large as has been seen for other plants. Our findings are in line with a report which documents that reduced lignin does not substantially improve the saccharification potential of transgenic poplars^55^, ascribed to complex biological aspect of plants which includes increase in phenolics as a consequence of perturbation of the phenylpropanoid pathway.

One remarkable observation of this study is that, different generations of both transgenic plant types showed no vital change in their phenotypic features (Table 1). This finding is of much significance, because the popularity of lignin engineering is frequently questioned due to observed growth retardation of transgenic plants with reduction in the percentage of their biomass^56^. For example in *Arabidopsis*^57^ *C4H* downregulation had resulted in dwarfism. In this respect, jute has been found to be more robust to lignin perturbation, emphasizing its potential industrial applications.

## Conclusion

In an earlier study, we had used artificial miRNA for manipulating the lignin pathway in order to reduce lignin content in jute by targeting two genes (coumarate 3- hydroxylase, *C3H* and ferulate 5-hydroxylase, *F5H*) different from the ones used in this study^58^. In the present study we have independently downregulated two other genes of the lignin biosynthetic pathway (*COMT* and *C4H*) by RNAi. Results show significant downregulation of transcripts as well as lignin content in the transgenic lines with an associated increase in cellulose and slight improvement in digestibility. UV fluorescence microscopy of stem cross sections of transgenic lines corroborate the biochemical estimation of lignin content. Even though digestibility testing does not show much improvement in release of sugars as compared to other transgenic plants, it supports the conventional theory of S/G ratio with respect to effective enzymatic saccharification. It is also possible that plants with different genetic make-ups have different responses to the same manipulation^22^. These data suggest that downregulation of lignin biosynthetic genes may lower the lignin content but the consequences of decreased lignin may not be the same in all modified plants. Discrepancies in the significances of such modifications appear to be rife.

## Methods

### Plant material

Seeds of *Corchorus olitorius* (var. 0–9897), one of the two cultivated jute species, were collected from the Physiology Department, Bangladesh Jute Research Institute (BJRI), Dhaka.

### Gene identification approach

Full length sequences of the two genes (*C4H* and *COMT*) used in the study were retrieved by using PCR based walking method. At first, parts of the two genes were identified using degenerate primers designed from conserved domains of the same genes from different plants followed by PCR amplifications, TA cloning (Invitrogen, USA) of the amplicons according to the manufacturer’s instruction, and sequencing. On the basis of the first sequence data, jute specific primers for particular genes were designed and gene walking approach was then followed from subsequent sequence data to extract the whole sequence of the two genes.

### hpRNAi constructs

Once the sequence was known, RNAi primers were designed from the conserved coding region to amplify the products. *COMT*-RNAi and *C4H*-RNAi products were amplified (primer sequences can be found in Supplementary Table-1) followed by TA vector mediated cloning to determine the RNAi amplicons (Supplementary Fig. 2). Then these amplicons were incorporated into pENTER11 plasmids and the constructs were confirmed by colony PCR and restriction digestion with enzymes (*Bam*HI/*Xho*I), the restriction sites for which are present in the constructs. Finally, the recombinant GATEWAY™ pENTER11 plasmids (*COMT*-pENTER11 and *C4H*-pENTER11) were mobilized into GATEWAY™ pK7GWIWG (II)^59^ destination vector using LR reaction (Supplementary Fig. 3.1, 3.2) in order to introduce the hairpin construct into the same.

### *Agrobacterium* mediated *in-planta* transformation

The hairpin constructs were then introduced into *C. olitorius* var 0–9897 by using a tissue culture independent *Agrobacterium tumefaciens* mediated gene transformation method^21^. Shoot tips of young jute plants (15–20 cm in height) were pricked with a fine needle. After an hour, the injured region was infected with a few drops of *A. tumefaciens* suspension, having an O.D. of 0.80 at 600 nm in YMB (Yeast Mannitol Broth) medium, containing the respective constructs. This was followed by a second infection in the same region an hour later. The infected plants were then incubated in dark for 3 days at 28 °C. After that the plants were grown under normal conditions of light.

### Genomic DNA PCR

The presence of the transgenes in the putative transformants (*COMT* -hpRNA lines and *C4H* –hpRNA lines) was assessed using genomic DNA from leaves of transformed (kanamycin-resistant) and non-transformed jute plants isolated by using the CTAB (N-Cetyl-N, N, N-trimethylammonium bromide) method. The presence of the reporter gene *NPT(II*), was identified by performing PCR using the primers, NPT (II) forward (5′-CCGTAAAGCACGAGGAAGTC-3′) and NPT (II) reverse (5′-ATGGGGATT GAACAAGATGG-3′). The PCR reaction included initial denaturation at 95 °C for 5 min followed by 30 cycles at 94 °C for 1 min, annealing at 56 °C for 30 s and extension at 72 °C for 30 s. The program ended with a final extension step for 7 min at 72 °C. Amplification products of approximately 350 bp were analyzed on a 0.8% agarose gel.

### Southern blot analysis

20 μg of genomic DNA from PCR-positive hpRNAi lines for *COMT* and *C4H* together with the wild type control were digested with *BamHI/Hin*dIII, electrophoresed and blotted on to Hybond N + membranes (GE Healthcare Life Sciences, UK) by overnight capillary transfer.

A 250 bp hpRNAi construct was used as a probe and purified in a G25 column (GE Healthcare Life Sciences, UK) according to the supplier’s protocol. The probes were labeled with [α^32P^] dCTP (PerkinElmer Life Sciences, USA). After hybridization for 20 h at 68 °C, the membrane was washed once with 2X saline-sodium citrate (SSC) containing 0.1% sodium dodecyl sulfate (SDS) at 60 °C for 20 min, then washed with 0.5X SSC containing 0.1% SDS at 60 °C for 30 min and finally washed with 2X SSC at room temperature and stored until exposure. The membranes were subjected to autoradiography using a TYPHOON phosphor imager (GE Healthcare Life Sciences, UK).

### Northern blot analysis

Total RNA was extracted from wild type and kanamycin screened 0–9897 jute seedlings of hpRNA transgenic lines (*COMT* & *C4H*) using the guanidium thiocynate extraction method. For northern blot of the transcripts 30 μg of total RNA from each plant sample was resolved on a 1.2% formaldehyde-agarose gel and transferred on to Hybond N + membranes (GE Healthcare Life Sciences, UK). The probes that spanned the entire coding sequence of the corresponding genes were prepared using [α^32P^] dCTP labelled cDNA of *COMT* and *C4H* genes individually.

For northern blot of hpRNA, total RNA was isolated from non-transgenic and transgenic hpRNA (*COMT* and *C4H*) lines using TRIZOL reagent and following the manufacturer’s protocol (Invitrogen). 20 μg of each sample was resolved on a 12% denaturing urea-PAGE gel. The RNA was then blotted onto a Hybond-N + membrane (GE Healthcare Life Sciences, UK) by Semi-Dry Transfer Cell (Bio-Rad Laboratories, Inc, CA). Probes were prepared using [α^32P^] dCTP labelled cDNAs that enclosed the respective hpRNAi amplicon specific sequences of *COMT* and *C4H* genes. Probes were purified in a G25 column (GE Healthcare Life Sciences, UK) according to the supplier’s protocol. Hybridization was carried out at 42 °C using a standard protocol. The membranes were subjected to autoradiography using a TYPHOON phosphor imager (GE Healthcare Life Sciences, UK).

### Reverse transcriptase polymerase chain reaction (RT-PCR) analysis

Approximately 2 μg of total RNA from non-transgenic and transgenic jute seedlings was used for first-strand cDNA synthesis, followed by gene specific PCR. cDNA was prepared in a 20 μL reaction volume, which contained 2 μL of reverse transcriptase (200 U/μL), using SuperScript^TM^ II reverse transcriptase kit (Invitrogen, USA) according to the manufacturer’s manual. The reaction was carried out with an initial incubation at 50 °C for 50 min, then 5 min at 85 °C to inactivate the enzyme. Next RNAse H was added followed by an incubation at 37 °C for 20 min to remove the cDNA-RNA hybrid. 1 μL of the cDNA sample was used as a template for PCR amplification with *COMT* GSP forward/*COMT* GSP reverse and *C4H* GSP forward /*C4H* GSP reverse primers (Supplementary Table 1) to amplify the respective samples. PCR of the same cDNA samples was carried out using gene-specific primers for actin to be used as a loading control in gel electrophoresis. All PCRs included 30 cycles of denaturation at 95 °C for 30 s, 40 s at the optimal temperature for each gene specific primer set (Supplementary Table 1) for annealing and 40 s at 72 °C for extension. RT-PCR band intensities were quantified using the ImageJ software (http://rsbweb.nih.gov/ij/index.html).

### Lignin content

Lignin content of jute whole stem and fiber was measured to determine the amount of acid insoluble lignin in jute for both transgenic and non-transgenic plants. To get the average lignin value of a plant, only the mid-section (~16 cm) of the stem was used in this study. A modified method of ribbon retting was followed for isolating the jute fibers^60^. Briefly, green ribbons (outer skin) of jute were stripped manually from barks of mature plants and submerged for 35 days in a tank in 1:10 ribbon to water ratio using water from the city’s regular supply. Next the fibers were washed and dried for downstream chemical measurements. A modified Klason lignin estimation method was used to estimate acid insoluble lignin or AIL as described by Tanmoy *et al*.^61^.

### Lignin composition

Lignin composition was determined from integration of the ^13^C-^1^H correlation signals in the 2D-NMR Heteronuclear Single Quantum Coherence (HSQC) of whole stems. Around 50 mg of finely divided (ball-milled) jute whole stem was swollen in 0.75 mL of Dimethyl sulfoxide-d6 (DMSO-*d*_6_) according to the method previously described^62^. 2D HSQC spectra were recorded at 25 °C on a Bruker AVANCE III 500 MHz instrument equipped with a cryogenically-cooled 5 mm TCI gradient probe with inverse geometry. 2D ^13^C-^1^H correlation spectra were carried out using an adiabatic HSQC pulse program (Bruker standard pulse sequence ‘hsqcetgpsisp2.2′) and the following parameters: spectra acquired from 10 to 0 ppm in F2 (1H) using 1000 data points for an acquisition time of 140 ms, an interscan delay of 1 s, and from 165 to 0 ppm in F1 (^13^C) using 256 increments of 32 scan, for a total acquisition time of 2 h 40 min. The ^1^*J*_CH_ used was 145 Hz. Processing used typical matched Gaussian apodization in ^1^H and a squared cosine bell in ^13^C. The central solvent peak was used as an internal reference (δ_C_/δ_H_ 39.5/2.49). 2D-NMR cross-signals were assigned by literature comparison. A semi quantitative analysis of the volume integrals of the HSQC correlation peaks was performed using Bruker’s Topspin 3.2 processing software. In the aromatic/unsaturated region, C_2_/H_2_ correlations from H, G, and S lignin units and from *p*-coumarates were used to estimate their relative abundances.

### Histochemistry and fluorescence microscopy

Lignified tissues were identified in the cross sections through histochemistry and fluorescence microscopy. Cross sectioning of jute stem samples was done using a microtome (Thermo Scientific™ HM 325) and sections were stained with phloroglucinol and observed under a light microscope (Nikon ECLIPES 50i) with a magnification of 100X. Presence of lignin was considered when the tissues were stained red. The fluorescence images were captured with a CCD (charged-couple device) camera.

### Cellulose content

Cellulose content of both wildtype and transgenic plants was determined using cold anthrone reagent^63^. Samples were taken from the whole stem. They were first digested by an 80% acetic acid: 100% nitric acid (8:1) solution and the precipitates were digested with 72% sulphuric acid. Addition of anthrone resulted in a green colored solution which gave an absorbance at 620 nm.

### Enzymatic saccharification

For enzymatic saccharification, first the neutral detergent fiber (NDF) was prepared^64^ and was then incubated for 24 h at 37 °C using commercial cellulase (Sigma, Source : *Aspergillus niger*) in 0.1 N sodium acetate buffer, pH 4.8, at 80 FPU/g NDF and measurement of reducing sugars was made by using dinitrosalicylic acid at 540 nm^65^.

### Statistical analysis

Five randomly selected plants of each generation of the selected lines were considered for phenotypical analyses. For lignin and cellulose content and enzymatic saccharification, two randomly selected biological replicates and technical triplicates of all three generations of each plant line for both transgenic plant types (CM P1-P2 and CH P1-P2) were taken into account. For lignin composition study, technical triplicates were taken from each plant line. Standard errors were calculated by using Microsoft excel tool pack 2013. For each parameter, the means, one way ANOVA test and Tukey’s test (for evaluating differences in each plant type) were performed using the R program. The alpha value was set at 0.001 (99.99% confidence). P value < 0.001 was considered as significant.

## Additional Information

**How to cite this article**: Shafrin, F. *et al*. Modification of Monolignol Biosynthetic Pathway in Jute: Different Gene, Different Consequence. *Sci. Rep.* **7**, 39984; doi: 10.1038/srep39984 (2017).

**Publisher's note:** Springer Nature remains neutral with regard to jurisdictional claims in published maps and institutional affiliations.

## Supplementary Material

Supplementary Information

## Figures and Tables

**Table 1:** Measurement of various growth and yield parameters of wild type and transgenic lines.

Parameters	wild type	*COMT*-hpRNA line			*C4H*-hpRNA line		
		T1	T2	T3	T1	T2	T3
Plant Height (cm)	335.24 ± 0.25^a^	334.84 ± 0.32^a^	334.7 ± 0.49^a^	335.52 ± 0.32^a^	334.8 ± 0.31^a^	335.5 ± 0.32^a^	335.4 ± 0.4^a^
Plant width (cm)	1.188 ± 0.02^a^	1.18 ± 0.003^a^	1.18 ± 0.0003^a^	1.19 ± 0.004^a^	1.22 ± 0.003^a^	1.19 ± 0.008^a^	1.20 ± 0.005^a^
Pod Number	61.8 ± 1.06^ab^	58 ± 0.44^c***^	60 ± 0.44^abc^	59 ± 0.31^bc^	62 ± 0.31^a^	60 ± 0.31^abc^	58 ± 0.31^c***^
Pod Length (cm)	5.00 ± 0.007^a^	4.9 ± 0.005^a^	5.00 ± 0.003^a^	4.9 ± 0.003^a^	4.9 ± 0.005^a^	4.9 ± 0.005^a^	5.00 ± 0.004^a^

Results are given as means ± standard error. Sample size = 5. Statistical analyses were done using one way ANOVA and Tukey’s test with P value < 0.001 considered as highly significant. (P value is given in Supplementary Table 2). ‘***’denotes P < 0.001, ‘**’denotes P < 0.01. For each individual parameter, means that do not share a letter are significantly different. (cm = centimeter).

**Table 2:** A summary of lignin content and composition, cellulose content, enzymatic glucose release and a comparative analyses between wild type and transgenic jute plants.

Parameters	Wildtype	*COMT*-hpRNA lines						*C4H*-hpRNA lines					
		P1			P2			P1			P2		
		T1	T2	T3	T1	T2	T3	T1	T2	T3	T1	T2	T3
% Klason lignin (whole stem)	29.50 ± 0.04^a^	25.61 ± 0.10^b***^	24.22 ± 0.21^c***^	23.81 ± 0.13^c***^	25.35 ± 0.16^b***^	23.97 ± 0.16^c***^	23.95 ± 0.14^c***^	22.90 ± 0.12^d***^	22.85 ± 0.15^d***^	22.96 ± 0.07^d***^	22.85 ± 0.14^d***^	22.56 ± 0.07^d***^	22.79 ± 0.16^d***^
% Lignin reduction (whole stem)		13.18	17.90	19.28	14.08	18.76	18.82	22.36	22.56	22.17	22.54	23.54	22.73
% Klason lignin (fiber)	13.46 ± 0.02^a^	11.61 ± 0.12^bcd***^	11.68 ± 0.14^bcd***^	11.54 ± 0.17^bcd***^	11.72 ± 0.16^bc***^	11.58 ± 0.11^bcd***^	11.86 ± 0.06^b***^	11.05 ± 0.18^d***^	11.74 ± 0.18^bc***^	11.97 ± 0.09^b***^	11.71 ± 0.13^bc***^	11.16 ± 0.07 ^cd***^	11.41 ± 0.13^bcd***^
% Lignin reduction (fiber)		13.72	13.24	14.25	12.91	13.97	11.86	17.89	12.82	11.07	13.03	17.07	15.23
% Cellulose (whole stem)	30.37 ± 0.13^d^	31.55 ± 0.14^abc***^	31.55 ± 0.05^abc***^	31.49 ± 0.11^abc***^	31.79 ± 0.13^a***^	31.87 ± 0.02^a***^	31.70 ± 0.07^ab***^	31.18 ± 0.16^c***^	31.23 ± 0.06^bc***^	31.58 ± 0.15^abc***^	31.45 ± 0.05^abc***^	31.42 ± 0.07^abc***^	31.64 ± 0.05^abc***^
% Increase in cellulose content (whole stem)		3.88	3.89	3.69	4.68	4.95	4.39	2.67	2.84	3.99	3.56	3.45	4.19
Ratio of cellulose to lignin	1.00 ^f^	1.23^e***^	1.30^d***^	1.32^bcd***^	1.25^e***^	1.33^bcd***^	1.36^ab***^	1.37^a***^	1.37^a***^	1.38^a***^	1.38^a***^	1.39^a***^	1.39^a***^
Amount of glucose released (mg/g of sample)	76.87 ± 0.31^b^	76.01 ± 0.63^b^	76.11 ± 0.55^b^	76.01 ± 0.63^b^	76.04 ± 0.61^b^	76.06 ± 0.51^b^	76.06 ± 0.47^b^	78.46 ± 0.23^a***^	78.18 ± 0.16^ab***^	78.36 ± 0.18^a***^	78.38 ± 0.22^a***^	78.03 ± 0.28^ab***^	78.37 ± 0.17^a***^
H (%)	0	0	0	0	0	0	0	0	0	0	0	0	0
S (%)	52 ± 0.57^e^	47.33 ± 0.67 ^f***^	46.67 ± 0.33 ^f***^	51.67 ± 0.88^e^	46.67 ± 0.33 ^f***^	47.33 ± 0.33 ^f***^	50.67 ± 0.88^e^	61.67 ± 1.20^a***^	55.33 ± 0.67 ^cd***^	53.33 ± 0.33^d***^	59.00 ± 0.58^ab***^	57.00 ± 0.58^bc***^	52.67 ± 0.33^d***^
G (%)	48.00 ± 0.58^b^	52.67 ± 0.67^a***^	53.33 ± 0.33^a***^	48.33 ± 0.88^b^	53.33 ± 0.33^a***^	52.67 ± 0.33^a***^	49.33 ± 0.88^b^	38.33 ± 1.20 ^f***^	44.67 ± 0.67 ^cd***^	46.67 ± 0.33^bc***^	41.00 ± 0.58^ef***^	43.00 ± 0.58^de***^	47.33 ± 0.33^bc***^
S/G=	1.1^e^	0.9^e^	0.9^e^	1.1^e^	0.9^e^	0.9^e^	1.1^e^	1.8^a***^	1.2 ^cd***^	1.2 ^cd***^	1.5^ab***^	1.4^bc***^	1.1^e^
*P* coumarate (%)^*^	11.33 ± 1.02^c^	9.33 ± 1.76^d**^	4.66 ± 0.57 ^g**^	9 ± 0.57^de**^	8.95 ± 0.81^e**^	5.78 ± 0.95 ^g**^	9.21 ± 1.05^d**^	15.7 ± 2.19^a**^	11.67 ± 0.88^c^	8.33 ± 2.19^ef**^	14.78 ± 1.56^b**^	11.12 ± 1.72^c^	9.26 ± 2.06^d**^

The sample size was six for lignin measurement, cellulose content and glucose release estimations including two biological replicates and technical triplicates. Three replicates were used for analyses of lignin composition. Results are given as means ± standard error. Statistical analyses were done using one way ANOVA and Tukey’s test with P value < 0.001 considered as highly significant (P value is given in Supplementary Table 2). ‘***’denotes P < 0.001, ‘**’denotes P < 0.01. For each individual parameter, means that do not share a letter are significantly different. *P* coumarate content expressed as a fraction of the total lignin aromatic units (H + G + S).
