# Supplementary material for: Modification of Monolignol Biosynthetic Pathway in Jute: Different Gene, Different Consequence
Source: Sci Rep. 2017 Jan 4;7:39984. doi: 10.1038/srep39984 (PMC5209690; doi:10.1038/srep39984)
Supplement: Supplementary Information [file srep39984-s1.pdf]

Modification of Monolignol Biosynthetic Pathway in Jute: Different Gene, Different Consequence

Farhana Shafrin [1], Ahlan Sabah Ferdous[1] , Suprovath Kumar Sarkar[1], Rajib Ahmed[1], Al- Amin[1], Kawsar Hossain[1], Mrinmoy Sarker[1], Jorge Rencoret[2], Ana Gutiérrez[2], Jose C. del Rio[2], Neeti Sanan-Mishra[3], Haseena Khan[1]*

```
ctagagggcccgacgtcgcatgcctgcaggtcactggattttggttttaggaattagaaattttattgataga
agtattttacaaatacaaatacatactaagggtttcttatatgctcaacacatgagcgaaaccctataagaac
cctaattcccttatctgggaactactcacacattattctggagaaaaatagagagagatagatttgtagagag
agactggtgattttgcggactctagcatggccgcgggatatcacaagtttgtacaaaaaagctgaacgagaa
acgtaaaatgatataaatatcaatatattaaattagatttgcataaaaaacagactacataatactgtaaaa
cacaacatatccagtcactatgATGATATCCCCGCTGAGAGCAAAATCTTGGTCAATGCATGGTGG
CTCGCCAACAACCCTGCCCAGTGGAAAAACCCCCAGGAGTTTAGGCCCGAGAGGTTCTTTGAA
GAGGAATCTAAGGTTGAGGCCAATGGCAATGACTTCAGGTACCTTCCATTTGGGGTTGGAAGAA
GAAGTTGCCCTGGAATTATTCTCGCTTTGCCCATCCTTGGTATcatagtgactggatatgttgtgttt
tacagtattatgtagtctgtttttatgcaaaatctaatttaatatattgatatttatatcattttacgtttc
tcgttcagctttcttgtacaaagtggtgatatcactagtgcggccgcctgcaggtcgaccatatggtcgacct
gcaggcggccgcactagtgatgctgttatgttcagtgtcaagctgacctgcaaacacgttaaatgctaagaag
ttagaatatatgagacacgttaactggtatatgaataagctgtaaataaccgagtataaactcattaactaat
atcacctctagagtataatataatcaaattcgacaatttgactttcaagagtaggctaatgtaaaatctttat
atatttctacaatgttcaaagaaacagttgcatctaaacccctatggccatcaaattcaatgaacgctaagct
taatatgactctcaataaagtctcataccaacaagtgccaccttattcaaccatcaagaaaaaagccaaaatt
tatgctactctaaggaaaacttcactaaagaagacgatttagagtgttttaccaagaatttctgtcatcttac
taaacaactaaagatcggtgtgatacaaaacctaatctcattaaagtttatgctaaaataagcataattttac
ccactaagcgtgaccagataaacataactcagcacaccagagcatatatattggtggctcaaatcatagaaac
ttacagtgaagacacagaaagccgtaagaagaggcaagagtatgaaaccttacctcatcatttccatgaggtt
gcttctgatcccgcgggatatcaccactttgtacaagaaagctgaacgagaaacgtaaaatgatataaatatc
aatatattaaattagatttgcataaaaaacagactacataatactgtaaaacacaacatatccagtcactat
gATACCAAGGATGGGCAAAGCGAGAATAATTCCAGGGCAACTTCTTCTTCCAACCCCAAATGGA
AGGTACCTGAAGTCATTGCCATTGGCCTCAACCTTAGATTCCTCTTCAAAGAACCTCTCGGGCC
TAAACTCCTGGGGGTTTTTCCACTGGGCAGGGTTGTTGGCGAGCCACCATGCATTGACCAAGA
TTTTGCTCTCAGCGGGGATATCATatagtgactggatatgttgtgttttacagtattatgtagtctgttt
tttatgcaaaatctaatttaatatattgatatttatatcattttacgtttctcgttcagctttttgtacaaa
cttgtgatatcactagtgcggccgcctgcaggtcgactagaatagtaaattgtaatgttgtttgttgtttgtt
ttgttgtggtaattgttgta
```

T35S

C4H-RNAi For

C4H-RNAi Rev

Intron R

intron_f

C4H-RNAi Rev

C4H-RNAi For

Figure 1.1: Sequence of C4H-RNAi construct

```
ctagagggcccgacgtcgcatgcctgcaggtcactggattttggttttaggaattagaaattttattgatagaagtat
tttacaaatacaaatacatactaagggtttcttatatgctcaacacatgagcgaaaccctataagaaccctaattccc
ttatctgggaactactcacacattattctggagaaaaatagagagagatagatttgtagagagagactggtgattttt
gcggactctagcatggccgcgggatatcacaagtttgtacaaaaaagctgaacgagaaacgtaaaatgatataaatat
caatatattaaattagatttgcataaaaaacagactacataatactgtaaaacacaacatatccagtcactatgACC
CAATTCACTCCAACTCAAGTCTCCGATGAGGAAGCAAACTTGTTCGCCATGCAATTGGCTAGTGCCTCAGTTCTTCCC
ATGGTCCTCAAATCTGCCATAGAACTTGACCTACTTGAAGTCATGGCCAAGGCTGGACCTGGTGCTTTCTTGTCCCCA
ACAGAAGTAGCTTCCCAATTGCCCACCAAGAACCCTGATGCACCCGTCATGCTCGACCGTATCTTGCGGCTCCTTGCT
AGTTACTCCATTTTAACTTGCTCCTTAAGGAATCTTCCTGATGGCAAAGTTGAGAGGCTCTATGGCCTTGGCCCTGTC
TGTAAATACCTGGTCAAGAATGAAGATGGTGTCGCTCTTTCCGCCCTTAATCTCcatagtgactggatatgttgtgtt
ttacagtattatgtagtctgtttttatgcaaaatctaatttaatatattgatatttatatcattttacgtttctcgt
tcagctttcttgtacaaagtggtgatatcactagtgcggccgcctgcaggtcgaccatatggtcgacctgcaggcggc
cgcactagtgatgctgttatgttcagtgtcaagctgacctgcaaacacgttaaatgctaagaagttagaatatatgag
acacgttaactggtatatgaataagctgtaaataaccgagtataaactcattaactaatatcacctctagagtataat
ataatcaaattcgacaatttgactttcaagagtaggctaatgtaaaatctttatatatttctacaatgttcaaagaaa
cagttgcatctaaacccctatggccatcaaattcaatgaacgctaagcttaatatgactctcaataaagtctcatacc
aacaagtgccaccttattcaaccatcaagaaaaaagccaaaatttatgctactctaaggaaaacttcactaaagaaga
cgatttagagtgttttaccaagaatttctgtcatcttactaaacaactaaagatcggtgtgatacaaaacctaatctc
attaaagtttatgctaaaataagcataattttacccactaagcgtgaccagataaacataactcagcacaccagagca
tatatattggtggctcaaatcatagaaacttacagtgaagacacagaaagccgtaagaagaggcaagagtatgaaacc
ttacctcatcatttccatgaggttgcttctgatcccgcgggatatcaccactttgtacaagaaagctgaacgagaaac
gtaaaatgatataaatatcaatatattaaattagatttgcataaaaaacagactacataatactgtaaaacacaaca
tatccagtcactatgGAGATTAAGGGCGGAAAGAGCGACACCATCTTCATTCTTGACCAGGTATTTACAGACAGGGCC
AAGGCCATAGAGCCTCTCAACTTTGCCATCAGGAAGATTCCTTAAGGAGCAAGTTAAAATGGAGTAACTAGCAAGGAG
CCGCAAGATACGGTCGAGCATGACGGGTGCATCAGGGTTCTTGGTGGGCAATTGGGAAGCTACTTCTGTTGGGGACAA
GAAAGCACCAGGTCCAGCCTTGGCCATGACTTCAAGTAGGTCAAGTTCTATGGCAGATTTGAGGACCATGGGAAGAAC
TGAGGCACTAGCCAATTGCATGGCGAACAAGTTTGCTTCCTCATCGGAGACTTGAGTTGGAGTGAATTGGGTatagtg
actggatatgttgtgttttacagtattatgtagtctgtttttatgcaaaatctaatttaatatattgatatttatat
cattttacgtttctcgttcagctttttgtacaaacttgtgatatcactagtgcggccgcctgcaggtcgactagaat
agtaaattgtaatgttgtttgttgtttgttttgttgtggtaattgttgta
```

T35S

COMT RNAi For

COMT RNAi Rev

Intron R

intron_f

COMT RNAi Rev

COMT RNAi For

Figure 1.2: Sequence of COMT-RNAi construct

Supplementary Table 1: List of Primers

**Primer name**	**Sequence**	**Tm(^{o}C)**
COMT_Degenerate_for	5’-ATGRGTTCRACYGGTKAAAC-3’	60.0
COMT_Degenerate _rev	5’-TTATRTGYCAACARCTACACG-3’	
C4H_Degenerate_for	5’-ATGGRTCTYCTCTTCCTRGAG-3’	59.0
C4H_Degenerate_rev	5’-TTAARAACYGCGTGRCTTAC-3’	
COMT_GSP_FOR	5’-TACCCGGGATGGGTTCAACTGC-3’	60.0
COMT_GSP_REV	5’-TGGAGCTCTTAAACAGTTTTGAGGAACTC-3’	
C4H_GSP_FOR	5’-ATCCCGGGATGGATCTTCTCTTCCTGGAG-3’	58.0
C4H_GSP_REV	5’-ATGAGCTCTTAAAAACTGCGTGGCTTAC-3’	
C4H-RNAi_For	5’- ATGATATCCCCGCTGAGAGC-3’	58.0
C4H-RNAi_Rev	5’- GCTTTGCCCATCCTTGGTAT-3’	
COMT-RNAi_For	5’- ACCCAATTCACTCCAACTCAAG-3’	59.0
COMT-RNAi_Rev	5’-**C**GCTCTTTCCGCCCTTAATCTC-3’	
Intron F	5’-GTCTCATACCAACAAGTGCCACCTT-3’	60.0
Intron R	5’-TGCATCTAAACCCCTATGGCCA-3’	
Actin For	5’-TGGTATTGGATGTTGGAGAT-3’	58.0
Actin Rev	5’-GGTATTGACTTAATGCTGCT-3’	

Supplementary Table 2: P values from one way ANOVA test for each parameters described in the text.

Alpha value = 0.05. Significance. Codes for P value: ‘***’ <0.001 ‘**’< 0.01 ‘*’< 0.05 ‘.’ <0.1 ‘ ’<1

**Parameters**	**P value**
**Plant height**	0.363
**Plant width**	0.0371.
**Pod number**	1.64e-08 ***
**Pod length**	0.058.
**% Klason lignin (whole stem)**	<2e-16 ***
**% Klason lignin (fiber)**	<2e-16 ***
**% Cellulose (whole stem)**	5.65e-14 ***
**Ratio of cellulose to lignin**	<2e-16 ***
**Amount of glucose released (mg/g of sample)**	8.11e-05 ***
**S (%)**	1.95e-15 ***
**G (%)**	1.95e-15 ***
**S/G=**	2.38e-14 ***
***P* Coumarates (%)**[*]	0.00493 **

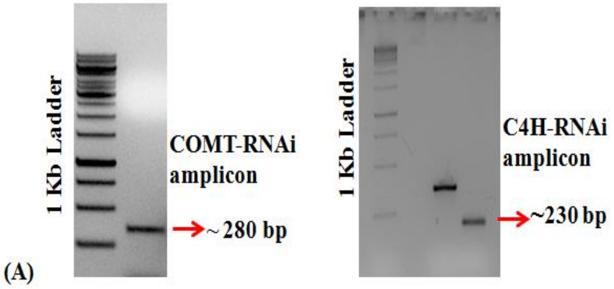

Figure 2: Agarose gel electrophoreses illustrates the RNAi-primer based amplification of COMT and C4H gene

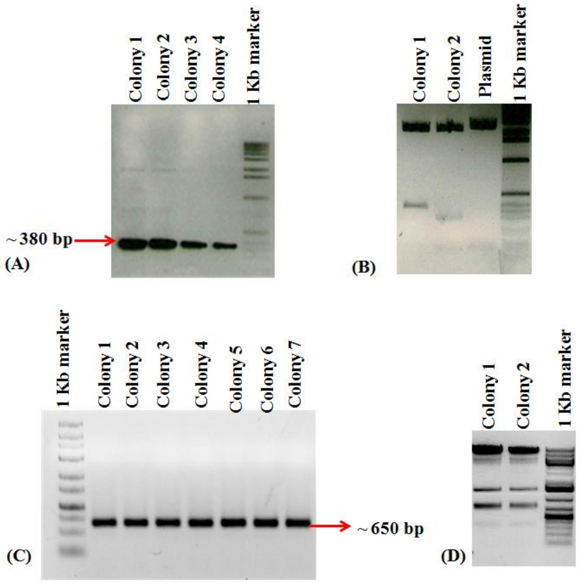

Figure 3.1: Represents the cloning confirmation for COMT-RNAi. (A) Colony PCR from pENTER11 (B) COMT-RNAi insert release from pENTER11 (C) Colony PCR from destination vector pK7GWIWG (II) (D) Insert release from destination vector upon restriction digestion with *BamHI*/*XhoI*.

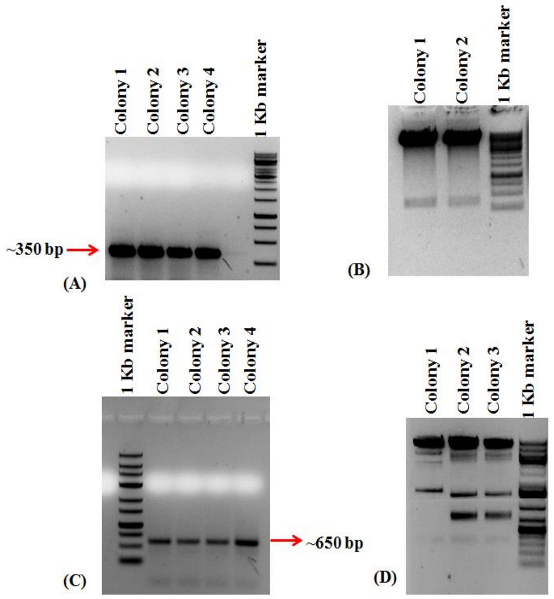

Figure 3.2: Shows the cloning confirmation for C4H-RNAi. (A) Colony PCR from pENTER11 (B) C4H-RNAi insert release from pENTER11 (C) Colony PCR from destination vector pK7GWIWG (II) (D) Insert release from destination vector upon restriction digestion with *BamHI*/*XhoI*
